# High Molecular Weight Forms of Mammalian Respiratory Chain Complex II

**DOI:** 10.1371/journal.pone.0071869

**Published:** 2013-08-13

**Authors:** Nikola Kovářová, Tomáš Mráček, Hana Nůsková, Eliška Holzerová, Marek Vrbacký, Petr Pecina, Kateřina Hejzlarová, Katarína Kľučková, Jakub Rohlena, Jiri Neuzil, Josef Houštěk

**Affiliations:** 1 Department of Bioenergetics, Institute of Physiology Academy of Sciences of the Czech Republic, Prague, Czech Republic; 2 Laboratory of Molecular Therapy, Institute of Biotechnology Academy of Sciences of the Czech Republic, Prague, Czech Republic; 3 Apoptosis Research Group, School of Medical Science and Griffith Health Institute, Griffith University, Southport, Queensland, Australia; UMASS-Amherst/Tufts University School of Medicine, United States of America

## Abstract

Mitochondrial respiratory chain is organised into supramolecular structures that can be preserved in mild detergent solubilisates and resolved by native electrophoretic systems. Supercomplexes of respiratory complexes I, III and IV as well as multimeric forms of ATP synthase are well established. However, the involvement of complex II, linking respiratory chain with tricarboxylic acid cycle, in mitochondrial supercomplexes is questionable. Here we show that digitonin-solubilised complex II quantitatively forms high molecular weight structures (CII_hmw_) that can be resolved by clear native electrophoresis. CII_hmw_ structures are enzymatically active and differ in electrophoretic mobility between tissues (500 – over 1000 kDa) and cultured cells (400–670 kDa). While their formation is unaffected by isolated defects in other respiratory chain complexes, they are destabilised in mtDNA-depleted, rho0 cells. Molecular interactions responsible for the assembly of CII_hmw_ are rather weak with the complexes being more stable in tissues than in cultured cells. While electrophoretic studies and immunoprecipitation experiments of CII_hmw_ do not indicate specific interactions with the respiratory chain complexes I, III or IV or enzymes of the tricarboxylic acid cycle, they point out to a specific interaction between CII and ATP synthase.

## Introduction

The mitochondrial oxidative phosphorylation system (OXPHOS) is the main source of energy in mammals. This metabolic pathway is localised in the inner mitochondrial membrane (IMM) and includes the respiratory chain complexes I, II, III and IV (CI, CII, CIII, CIV), ATP synthase (complex V, CV), plus the mobile electron transporters coenzyme Q (CoQ) and cytochrome *c*. Energy released by oxidation of NADH and FADH_2_ is utilised for proton transport across the membrane to establish proton gradient. The resulting electrochemical potential (Δμ_H_^+^) is then utilised as a driving force for phosphorylation of ADP by ATP synthase.

CII (succinate: ubiquinone oxidoreductase; EC 1.3.5.1), catalyses electron transfer from succinate (via FADH_2_) to CoQ and thus represents important crossroads of cellular metabolism, interconnecting the tricarboxylic acid (TCA) cycle and the respiratory chain [1]. It consists of 4 nuclear encoded subunits. The hydrophilic head of CII is formed by the SDHA subunit with covalently bound FAD and the SDHB subunit, which contains three Fe–S centres. The SDHC and SDHD subunits form the hydrophobic membrane anchor and are the site of cytochrome *b* binding [2].

Mutations in genes coding for any of the CII subunits are associated with severe neuroendocrine tumours such as paraganglioma and phaeochromocytoma [3–5] as well as other tumour types, including gastrointestinal stromal tumours [6] or renal tumours [7]. Conversely, the CII subunits also function as tumour suppressors and represent one of the potential molecular targets of anti-cancer drugs [8], whose mechanisms of action could lead to apoptosis of cancer cells through the inhibition of CII and a consequent metabolic collapse.

In comparison with other respiratory chain complexes, the assembly of CII has not yet been fully characterised. Up to now, two evolutionarily conserved assembly factors for CII have been described; SDHAF1 was discovered as disease-causing gene in a case of infantile leukoencephalopathy presenting with a decrease in the CII content and activity [9]. The LYR motif in the protein structure suggests its role in the metabolism of the Fe–S centres [10]. The second assembly factor, SDH5, is a soluble mitochondrial matrix protein, which is most likely required for insertion of FAD into the SDHA subunit [11].

Recent studies indicate that the organisation of the OXPHOS complexes in the inner mitochondrial membrane (IMM) is characterised by non-stochastic protein–protein interactions. Individual complexes specifically interact with each other to create supramolecular structures referred to as supercomplexes (SCs). SCs behave as individual functional units, enabling substrate channelling [12]; more effective electron transport should prevent electron leak and reactive oxygen species generation [13]. Besides the kinetic advantage, SCs stabilise OXPHOS complexes and help to establish the IMM ultrastructure [14].

To date, the presence of CII in SCs is still a matter of debate. In yeast and mammalian mitochondria, the interaction of CI, III, IV and V within different types of SCs has been proven using native electrophoretic techniques in combination with mild detergents and/or the Coomassie Blue G (CBG) dye [15,16]. However, the presence of CII in such structures has only been reported by Acín-Peréz et al. [17], who described the existence of a large respirasome comprising all OXPHOS complexes including CII in mammalian cells. On the other hand, CII has been detected as a structural component of the mitochondrial ATP-sensitive K^+^ channel (mitoK_ATP_) [18]. Such structures do indeed represent higher molecular forms of CII, but their structural and physiological importance remains to be investigated.

CII as the only membrane bound component of the TCA cycle could also form complexes with other TCA cycle proteins, e.g. with its functional neighbours fumarase and succinyl CoA lyase. Different studies indicate the existence of a TCA cycle metabolon and possible supramolecular organisation of various parts of the TCA cycle [19,20], but these may be significantly more labile than the well described respiratory chain SCs.

In the present study we demonstrate the existence of high molecular weight forms of CII (CII_hmw_), i.e. SCs containing CII, using mitochondrial membrane solubilisation with mild non-ionic detergents followed by electrophoretic analysis. These complexes are rather labile, and the presence of n-dodecyl-β-D-maltoside or CBG during the electrophoretic separation causes their dissociation to individual units. CII_hmw_ structures differ in their electrophoretic migration between mammalian cells and tissues, and their formation depends on the presence of the functional respiratory chain. Our experiments also clearly indicate the association of CII with CV.

## Materials and Methods

### Cell lines

The following cell lines were used in experiments: control human fibroblasts and fibroblasts from patients with isolated deficiency of CI (an unknown mutation), CIV (the *SURF1* mutation, described in [21,22]), CV (the *TMEM70* mutation described in [23]), human rho0 (ρ^0^) cells (mtDNA-depleted 143B TK^-^ osteosarcoma cells [24]), human embryonic kidney cells HEK293, primary mouse (derived from the C57/Bl6 strain) and rat (derived from the SHR strain) fibroblasts. All cell lines were grown in the high-glucose DMEM medium (Lonza) supplemented with 10% (v/v) foetal bovine serum (Sigma) at 37 °C in 5% CO_2_ atmosphere. Cells were harvested using 0.05% trypsin and 0.02% EDTA and stored as pellets at -80 °C.

### Isolation of cell membranes and mitochondria from cells and tissues

Mitochondria from cultured cells were isolated after cell disruption by hypotonic shock as described [25]. In some experiments, membrane fractions from fibroblasts were prepared as described [26]. Human heart mitochondria and mitochondria from rat heart, liver and brown adipose tissues were isolated according to established procedures [27]. The protein concentration was measured by the Bradford method (BioRad).

### Ethical aspects

All work involving human samples was carried out in accordance with the Declaration of Helsinki of the World Medical Association and was approved by the Ethics Committee of the Institute of Physiology, Academy of Sciences of the Czech Republic v.v.i. The written informed consent was obtained from patients or patients’ parents.

All animal tissues were obtained on the basis of approval by the Expert Committee for Work with Animals of the Institute of Physiology, Academy of Sciences of the Czech Republic v.v.i. (Permit Number: 165/2010) and animal work was in accordance with the EU Directive 2010/63/EU for animal experiments.

### Electrophoresis and western blot analysis

Isolated membranes or mitochondria were solubilised with digitonin (Sigma, 4 g/g protein) in an imidazole buffer (2 mM aminohexanoic acid, 1 mM EDTA, 50 mM NaCl, 50 mM imidazole, pH 7.0) for 15 min at 0 °C and centrifuged for 20 min at 20 000 *g* [26]. Samples were prepared by adding 5% (v/v) glycerol and 0.005% (v/v) Ponceau S dye for clear native and high resolution clear native electrophoresis (CNE, hrCNE3), or 5% (v/v) glycerol and CBG dye (Serva Blue G 250, 1:8 ratio (w/w) to digitonin) for blue native electrophoresis (BNE). Separation of mitochondrial proteins was performed using CNE, BNE [26] and hrCNE3 [28] on 6–15% polyacrylamide gradient gels using the Mini-Protean apparatus (BioRad). For 2D separation by CNE/SDS PAGE, the gel after CNE was cut into stripes that were incubated in 1% SDS and 1% 2-mercaptoethanol for 1 h and then subjected to SDS PAGE on a 10% slab gel [29]. In case of 2D separation by CNE/CNE_CBG_, gel stripes after CNE were incubated in 3% CBG in the CNE cathode buffer for 1 h and then subjected to CNE on 6–15% gradient gels.

For western blot immunodetection, the separated proteins were transferred to a PVDF membrane (Immobilon-P, Millipore) by semi-dry electrotransfer. The membranes were blocked with 5% (w/v) non-fat dried milk in TBS (150 mM NaCl, 10 mM Tris, pH 7.5) for 1 h and incubated overnight at 4 °C with specific primary antibodies diluted in TBST (TBS with 0.1% Tween-20). Monoclonal or polyclonal primary antibodies to the following enzymes of OXPHOS or TCA cycle were used: SDHA (ab14715, Abcam), SDHB (ab14714, Abcam), Core1 (ab110252, Abcam), NDUFA9 (ab14713, Abcam), Cox4 (ab14744, ab110261, Abcam), citrate synthase (ab129095, Abcam), isocitrate dehydrogenase (α subunit, ab58641, Abcam), aconitase 2 (ab110321, Abcam), α subunit of CV [30], fumarase (M01, Abnova), succinyl-CoA synthetase (α subunit, 5557, Cell Signaling Technology) and malate dehydrogenase (8610, Cell Signaling Technology). The detection of the signals was performed with the secondary Alexa Fluor 680-labelled antibody (Life Technologies) using the Odyssey fluorescence scanner (LI-COR).

### Enzyme in-gel activity staining

In-gel activity assays were performed after separation of the respiratory complexes using CNE. For CIV in-gel activity staining, we used a recently described protocol [21]. The in-gel activity assay of the CV ATP hydrolytic activity was performed as described [28]. The activity of CII was detected using the modified succinate: nitroblue tetrazolium reductase assay [28]. Briefly, gel slices from CNE were incubated for 1 h (for tissues) or overnight (for cells) at room temperature in the dark in the staining solution (200 mM Tris, pH 7.4), 10 mM EDTA, 1 mg/mL nitroblue tetrazolium, 80 µM phenazine methosulfate, 2 mM KCN, 1.5 µg/mL rotenone and 30 mM succinate).

### Immunoprecipitation

For co-immunoprecipitation analysis we used a rabbit polyclonal antibody against the F_1_ part of ATP synthase (reacting with the ATP synthase subunits α, γ, and predominantly β, generated in our laboratory) or a mouse monoclonal antibody against the SDHA subunit of CII (ab14715, Abcam). The antibodies were immobilised on CNBr-activated agarose matrix (Sigma). Agarose beads with the bound antibody were equilibrated in PBS (140 mM NaCl, 5 mM KCl, 8 mM Na_2_HPO_4_, 1.5 mM KH _2_PO_4_, pH 7.2 -7.3) supplemented with 0.2% protease inhibitor cocktail (PIC, Sigma). For storage at 4 °C, they were dissolved in PBS+PIC supplemented with 0.025% thimerosal (Sigma). Solubilisation of rat heart mitochondria and human fibroblasts was performed with digitonin (2 g/g protein) in PBS+PIC. The solubilisates were mixed with the antibody-conjugated agarose beads and diluted with PBS+PIC supplemented with the same digitonin concentration as for sample solubilisation. The mixture was incubated overnight at 4 °C on a rotating mixer. The beads were then washed three times with PBS+PIC+digitonin (the same concentration as for sample solubilisation), PBS+PIC+digitonin (ten times diluted), and finally with PBS+PIC. All the washing steps included incubation for 5 min at 4 °C on a rotating mixer and centrifugation at 1000 *g* for 1 min at room temperature. The pelleted beads were combined with a small volume of the 2x SDS sample lysis buffer and incubated at 65 °C for 15 min. After a brief centrifugation, the supernatant with the released co-immunoprecipitated proteins was subjected to SDS PAGE and western blot analysis using specific antibodies (described in section 2.4.).

## Results

### High molecular weight forms of CII

**Figure 1:**
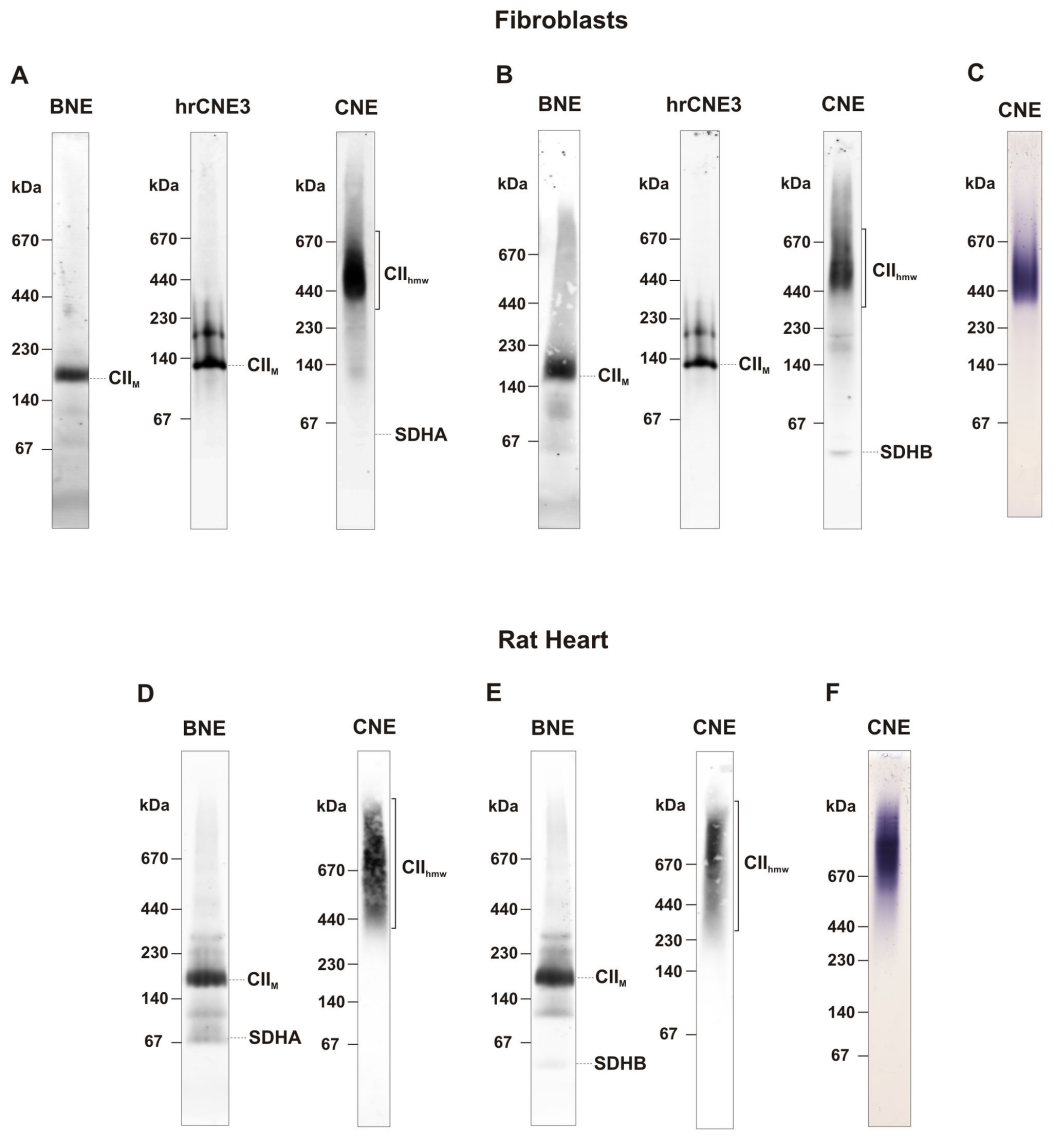
Higher molecular weight forms of complex II. Mitochondrial membrane proteins from control fibroblasts and rat heart were solubilised with digitonin (4 g/g protein), and 20 µg protein aliquots were separated using BNE, hrCNE3 and CNE. CII was immunodetected with the SDHA antibody (A, D) and SDHB antibody (B, E). In-gel activity staining of CII was performed in CNE gels (C, F). Migrations of higher molecular weight forms of CII (CII_hmw_), CII monomer (CII_M_), SDHA and SDHB subunits of CII are marked. The images are representative of three independent experiments.

**Figure 2:**
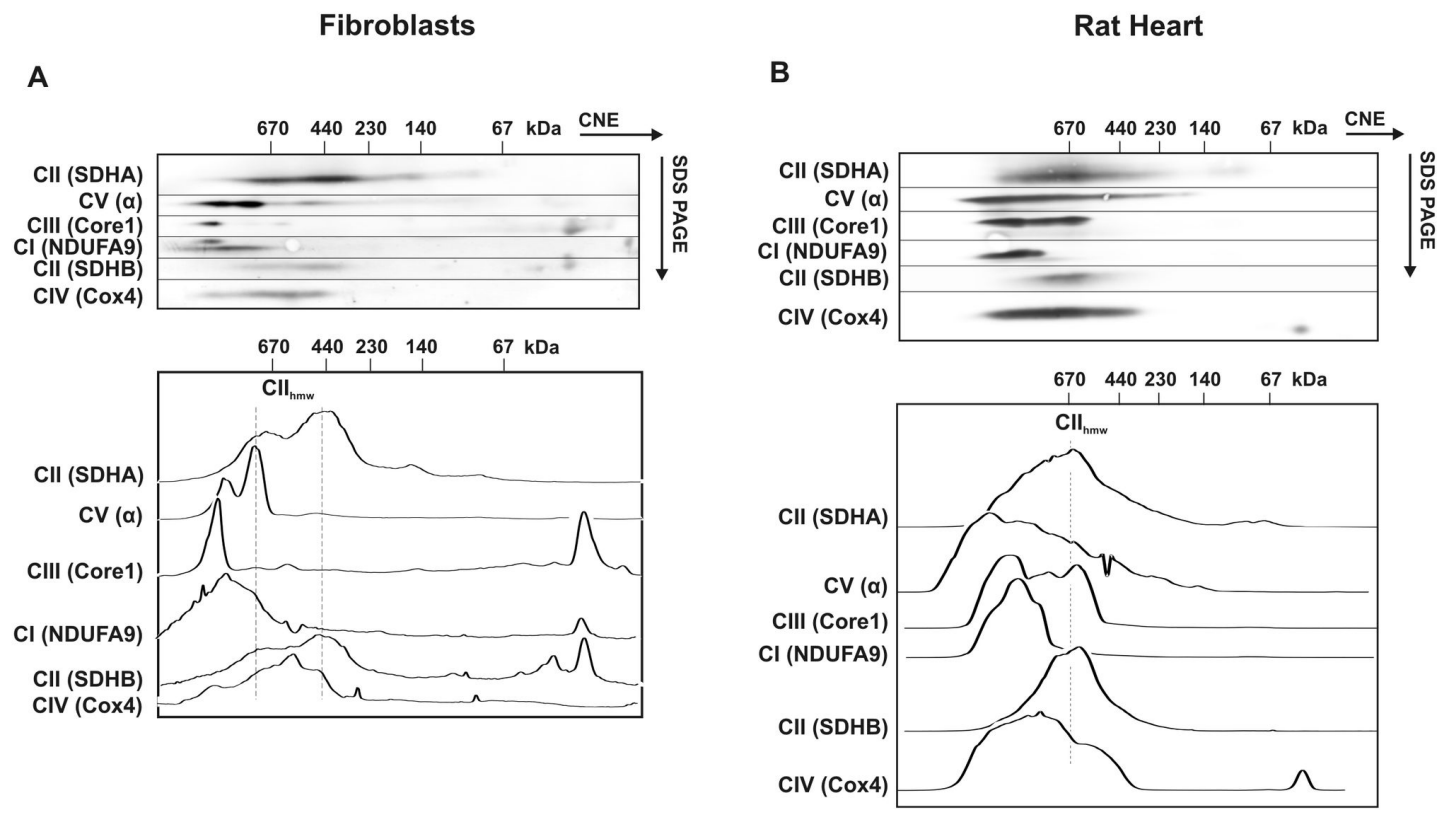
CNE/SDS PAGE analysis of OXPHOS proteins. Digitonin-solubilised proteins from human fibroblasts (A) and rat heart (B) mitochondria were separated by CNE in the first dimension (40 µg protein load) and by SDS PAGE in the second dimension. Subunits of the respiratory chain CI (NDUFA9), CII (SDHA, SDHB), CIII (Core1), CIV (Cox4) and CV (α) were immunodetected using specific antibodies. The dashed vertical lines in the distribution profiles below the western blots depict the main area of higher molecular weight forms of complex II (CII_hmw_).

The mammalian CII consists of four subunits, SDHA, SDHB, SDHC and SDHD, with the approximate molecular weight (MW) of 70, 30, 18 and 17 kDa, respectively. Digitonin-solubilised CII from mitochondria of human fibroblasts was resolved by BNE (in the presence of CBG) or hrCNE3 (in the presence of n-dodecyl-β-D-maltoside and deoxycholic acid in the cathode buffer) as a CII monomer of the expected mass of approximately 140 kDa (Figure 1A, B) which represented most of the CII signal. In addition, weaker bands smaller than 140 kDa and at approximately 200 kDa were also present; these could be CII sub-complexes and CII hetero-oligomers. When milder conditions of separation were applied using CNE, a completely different pattern of the CII signal was obtained, indicating the presence of its higher molecular weight forms (CII_hmw_). As revealed by immunodetection with the SDHA antibody, the signal of CII was almost completely localised within the region of 400–670 kDa (Figure 1A). Similarly, the SDHB antibody (Figure 1B) or in-gel staining of CII (SDH) activity (Figure 1C) confirmed the presence of CII in the 400–670 kDa region. This was further demonstrated by 2D CNE/SDS PAGE analysis of the cells (Figure 2A), where the distributions of SDHA and SDHB in the second dimension gel indicate that the CII_hmw_ forms represent a complete and active CII, in accord with the profiles of CII activity in CNE. For comparison, we also analysed the CII profile in mitochondria isolated from rat heart and obtained similar results, except for the size of tissue CII_hmw_ on CNE gels, which increased to 500 – over 1000 kDa when detected either with the SDHA or SDHB antibodies (Figure 1D, E) or by the in-gel SDH activity staining (Figure 1F). This was also confirmed by 2D analysis (Figure 2B).

Further, we analysed the distribution profiles of other OXPHOS complexes on 2D blots with subunit-specific antibodies in an attempt to determine potential CII interaction partners within CII_hmw_. As expected, a substantially different migration pattern was found in the case of CI (NDUFA9) while some CIII (Core1) signal overlapped with CII in heart, but not in fibroblasts (Figure 2A, B). The signal of CIV (Cox4) partially overlapped with that of CII_hmw_ (SDHA, SDHB), as shown by the distribution profiles below the western blot images. A similar overlap with the CII_hmw_ signal was found for CV (the α subunit), in particular in the case of fibroblasts (Figure 2A). This may reflect a coincidental co-migration of respective complexes because of the imprecise electrophoretic mobility inherent to the CNE system in the first dimension [31], but it can also indicate a possibility that CII_hmw_ include SCs of CII with CIII, CIV or CV.

### CII_hmw_ differ between tissues and cells

**Figure 3:**
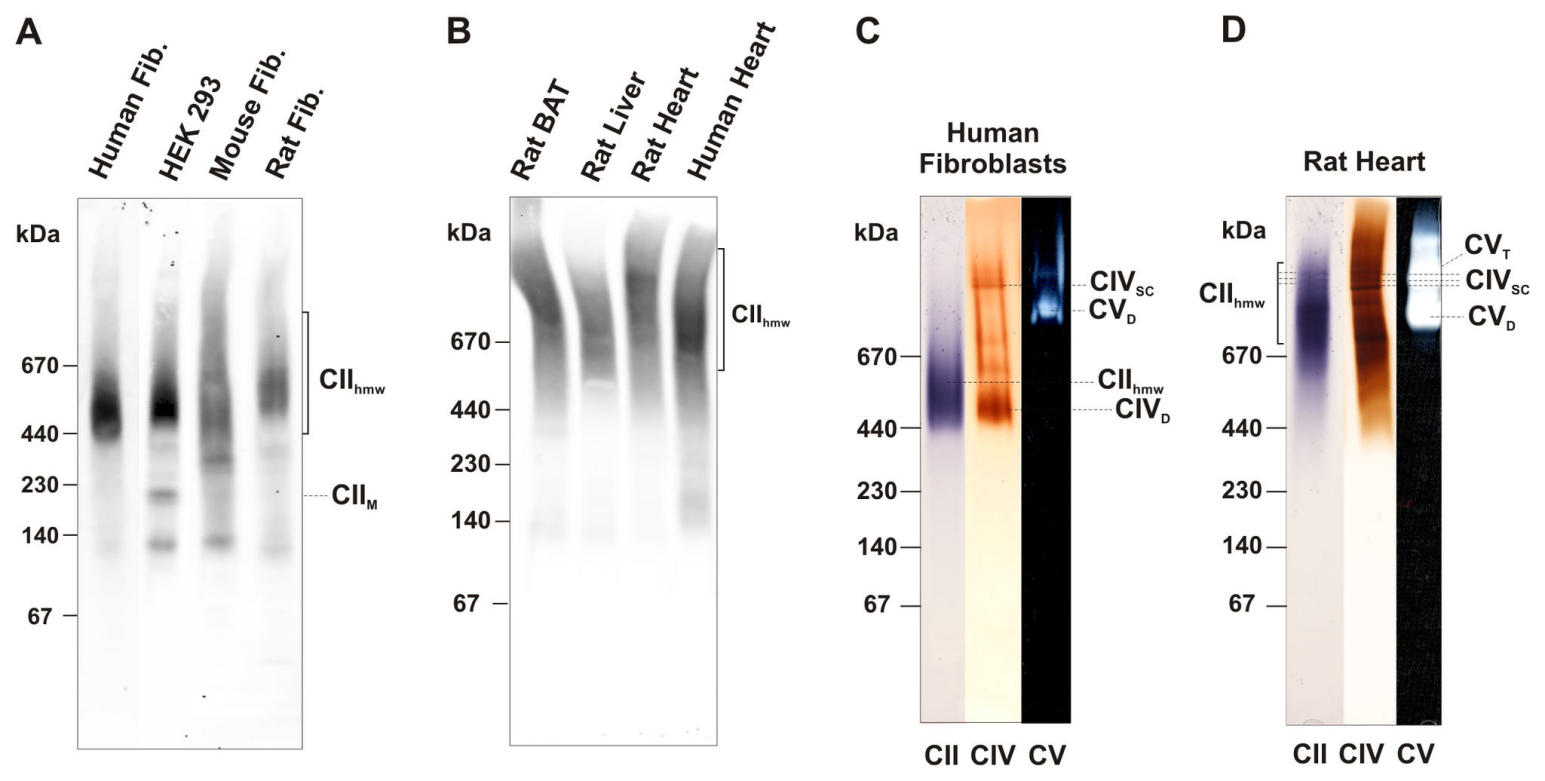
Comparison of CII_hmw_ in cells and tissues by western blot and in-gel activity staining. Mitochondria from different human and rodent cells (A) and tissues (B) were solubilised with digitonin (4 g/g protein) and 20 µg protein aliquots were separated using CNE. CII was immunodetected with the SDHA antibody. The activities of CII (violet), CIV (brown) and CV (white) in CNE gels (protein load 50 µg for cells and 40 µg for tissues) are shown in human fibroblasts (C) and rat heart (D). The positions of higher molecular weight forms of CII (CII_hmw_), CII monomer (CII_M_), CIV dimer (CIV_D_) and its supercomplexes (CIV_SC_), CV dimer (CV_D_) and tetramer (CV_T_) are indicated in the figure. Rat BAT, rat brown adipose tissue.

When CNE analysis of digitonin-solubilised proteins was performed using fibroblasts and different immortalised/malignant human or rodent cell lines (Figure 3A), an analogous CII_hmw_ pattern was obtained in all human, mouse and rat cells, indicating that most of CII is present as CII_hmw_. Similarly, CII_hmw_ was found as a predominant form of CII in mitochondria of different human and rodent tissues (Figure 3B), suggesting that high molecular forms of CII are a universal property of mammalian respiratory chain. Nevertheless, the mobility of CII_hmw_ in tissues was considerably different in comparison with cell lines. As shown in Figure 3B the main signal of the SDHA antibody in tissues was detected above 670 kDa, in the MW range of larger respiratory SCs (SDHB displayed an analogous distribution pattern, not shown). This was also observed on 2D CNE/SDS PAGE western blots (Figure 2B), where the signal of the CII SDHA and SDHB subunits was shifted to a higher MW. In contrast, other OXPHOS complexes were distributed comparably with the cultured cells (Figure 2A). Therefore, we performed in-gel activity staining of CII in CNE gels to confirm the detected antibody signals in the cells and tissues. Figure 3C, D reveals that CII_hmw_ complexes were catalytically active and, indeed, differed between cells and tissues. In parallel, we performed in-gel CIV and CV activity staining to further analyse a possible co-migration or interaction with CII. In the case of cells, the dominant CIV activity signal could be ascribed to the CIV dimer (CIV_D_) (Figure 3C), in the position corresponding to some of CII_hmw_. The higher active CIV SCs did not co-migrate with the CII signal. Thus, the size of CII_hmw_ in the cells more likely points to a mere co-migration of CII homo-/hetero-oligomers with CIV_D_, rather than to a genuine specific interaction between the OXPHOS complexes.

Interestingly, the CIV activity signals were shifted to the higher MW in tissues and overlapped with the activity signal of CII_hmw_ (see Figure 3D). The interaction of CII with CIV or other OXPHOS complexes in the MW range > 1 MDa thus cannot be excluded. The differences in the size of CII_hmw_ when comparing cells and tissues could suggest the existence of two major functional forms of CII SCs. In cells, they may be present largely as CII homo-oligomers, while in tissues, CII may possibly form SCs with other OXPHOS complexes. In-gel activity of monomeric and homo-oligomeric CV did indicate co-migration or interaction with CII_hmw_ in tissues but not in cells (Figure 3C, D).

### CII_hmw_ formation depends on other respiratory chain complexes

**Figure 4:**
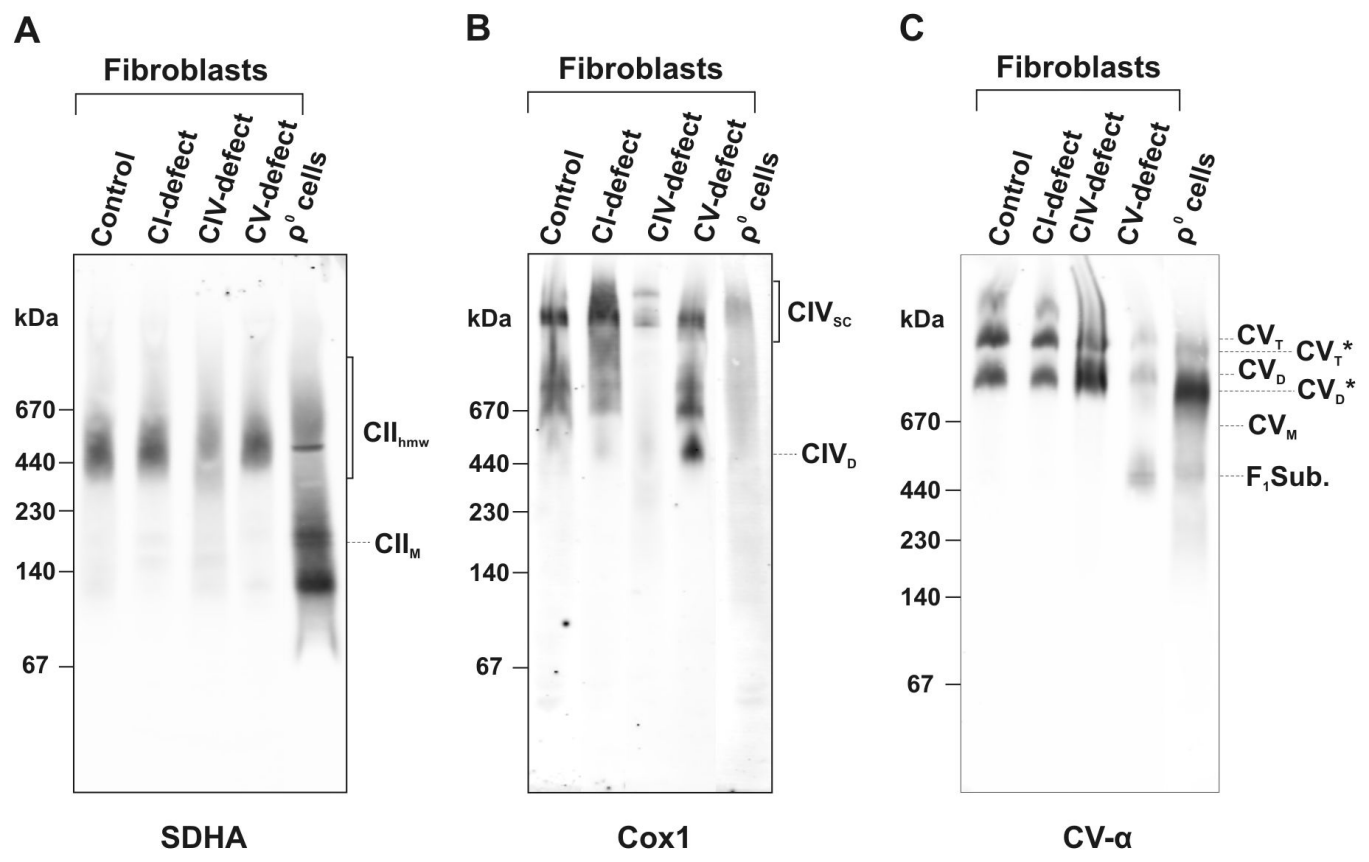
Presence of CII_hmw_ in human fibroblasts with different types of OXPHOS defects and ρ^0^ cells. Digitonin-solubilised mitochondrial complexes were analysed by CNE (20 µg protein load) and immunodetected using antibodies to individual subunits: (A) CII, SDHA; (B) CIV, Cox1; (C) CV, α subunit. Positions of the CII monomer (CII_M_), high molecular weight forms of CII (CII_hmw_), CIV dimer (CIV_D_), supercomplexes of CIV (CIV_SC_), F_1_ subcomplex of CV (F _1_Sub.), the monomer, dimer and tetramer of CV (CV_M_, CV_D_ and CV_T_), and the dimer and tetramer of CV lacking the mtDNA-coded subunits (CV_D_* and CV_T_*) are marked.

To learn more about possible interactions with other OXPHOS complexes, we performed CNE analysis of digitonin-solubilised mitochondria of human fibroblasts harbouring different types of OXPHOS defects that affect one or more respiratory chain complexes. We found that the selective deficiency of CIV (due to a *SURF1* mutation, Figure 4B) or CV (due to a *TMEM70* mutation, Figure 4C) did not affect the presence of CII_hmw_ (Figure 4A). Similarly, the selective deficiency of CI (an unknown mutation) was without any effect on the CII_hmw_ pattern. However, we obtained a different pattern in ρ^0^ cells with depletion of mtDNA and thus lack of functional complexes I, III, IV and V [24]. Here, most of the CII_hmw_ signal disappeared and CII was present as unassembled subunits or monomer. This demonstrates the requirement of fully assembled CII monomer for subsequent CII_hmw_ formation, and also its dependence on the preserved integrity of a fully functional respiratory chain (Figure 4A).

### CII_hmw_ stability depends on very weak protein–protein interactions

**Figure 5:**
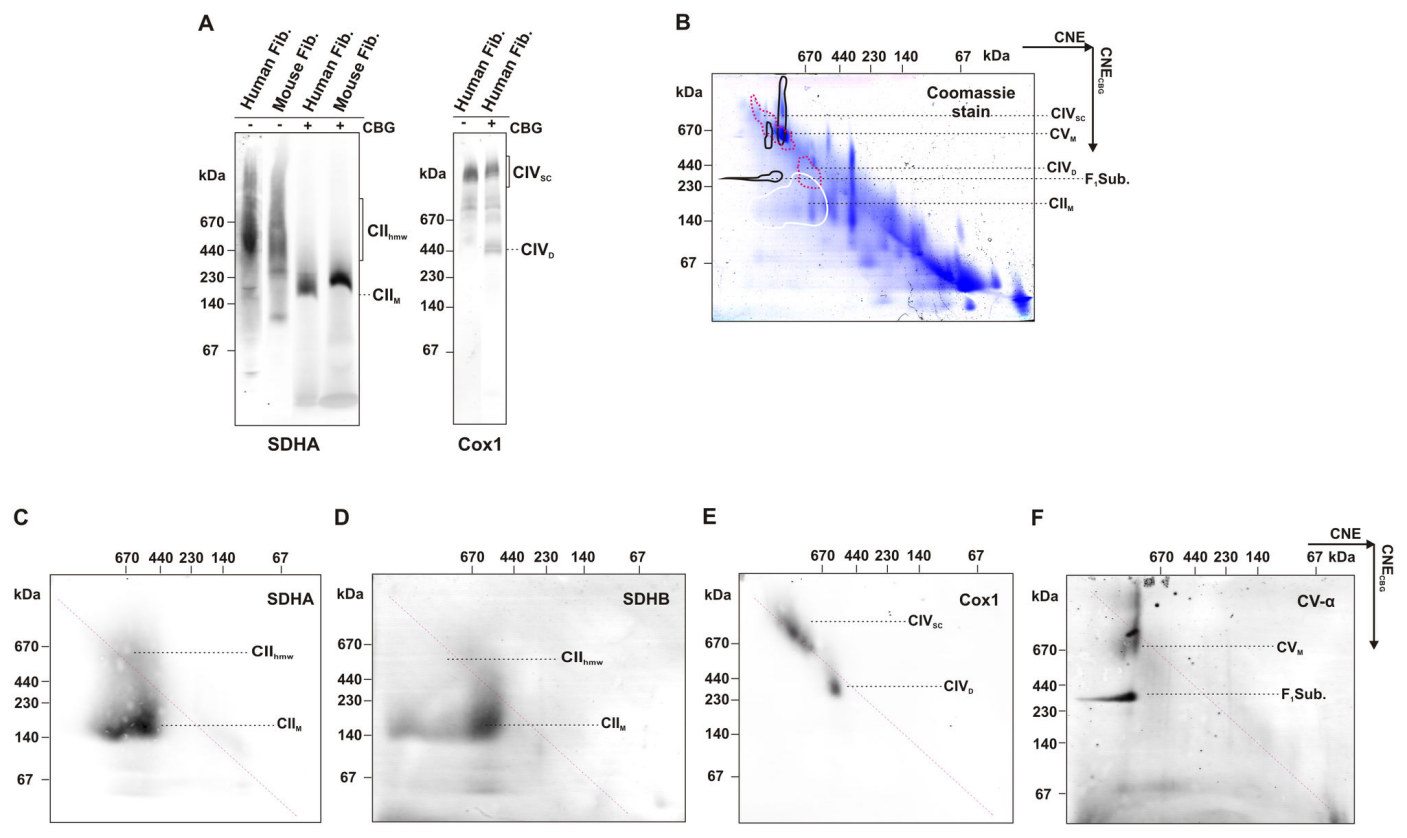
Low stability of CII_hmw_ in fibroblasts. (A) Digitonin-solubilised (4 g/g protein; 20 µg protein load) mitochondrial proteins from control human fibroblasts or control mouse fibroblasts were resolved by CNE with (+) or without (-) CBG. (B–F) Two-dimensional CNE/CNE_CBG_ analysis of mitochondrial proteins from control fibroblasts (50 µg protein load). After separation of mitochondrial proteins with CNE, the gel slices were incubated in CBG and subjected to CNE in the second dimension. One gel was stained in Coomassie blue stain and identical duplicate gel was used for western blot. The positions of individual OXPHOS complexes are highlighted on the stained gel (B) according to their immunodetection: full white line, CII monomer (CII_M_); dashed red line, CIV dimer (CIV_D_) and supercomplexes of CIV (CIV_SC_); full black line, F_1_ subcomplex of CV (F_1_Sub.) and monomer of CV (CV_M_) based on the signals of SDHA (C), SDHB (D), Cox1 (E) and CV-α (F) subunits.

The fact that CII_hmw_ are retained in CNE gels but dissociate in BNE gels (Figure 1) points to their rather labile nature. To analyse these interactions in more detail, we used CNE as before but with the CBG dye added to the sample (Figure 5A). In this experiment, CII_hmw_ dissociated into monomeric CII due to the presence of CBG, while other respiratory chain SCs (CIV shown as an example) remained unaffected, apart from the fact that they were better focused due to the negative charge introduced by CBG. We therefore performed 2D CNE/CNE_CBG_ electrophoresis using CBG to treat the gel slice after the CNE separation in the first dimension (Figure 5 B–F). As shown by western blots with the antibodies to SDHA and SDHB, all CII_hmw_ dissociated into CII monomers after exposure to CBG, that can bind to proteins due to its negative charge and thus interfered with weak non-covalent interactions. The main signal of CII from the first dimension can again be observed within the MW range of 400–670 kDa. On the contrary, the SCs of CI+III+IV were practically unaffected by CBG treatment (Figure 5E). Interestingly, the addition of CBG also partially affected oligomers of CV, which dissociated to lower molecular weight forms corresponding to the CV monomer and the F_1_ sub-complex (Figure 5F).

**Figure 6:**
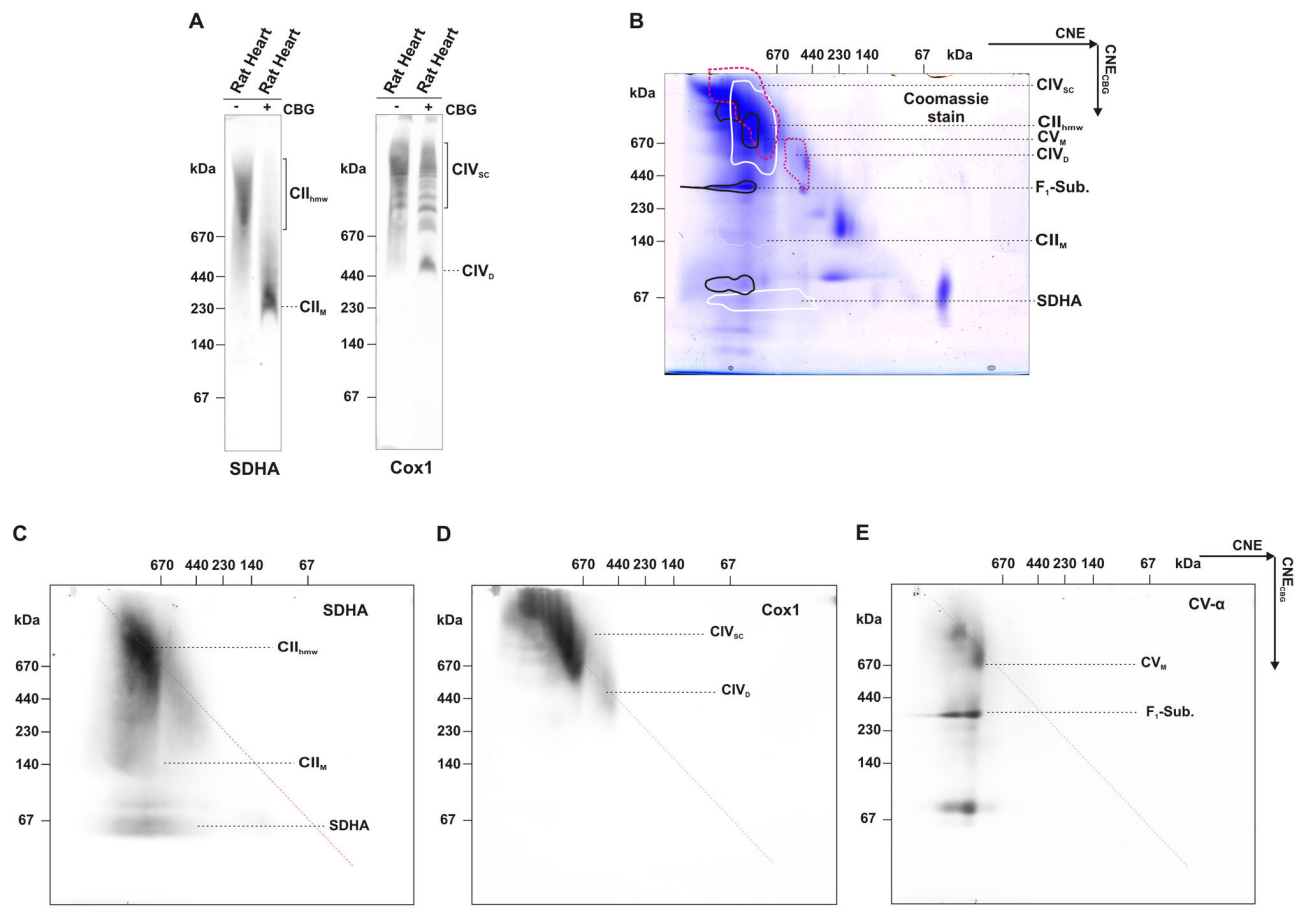
Low stability of CII_hmw_ in rat heart. (A) Digitonin-solubilised (4 g/g protein, 20 µg protein load) mitochondrial proteins from rat heart mitochondria were resolved by CNE with (+) or without (-) CBG. (B–F) Two-dimensional CNE/CNE_CBG_ analysis of mitochondrial proteins from rat heart (40 µg protein load). After separation of mitochondrial proteins with CNE, the gel slices were incubated in CBG and subjected to CNE in the second dimension. One gel was stained in Coomassie blue stain and identical duplicate gel was used for western blot. The positions of individual OXPHOS complexes are highlighted on the stained gel (B) according to their immunodetection: full white line, CII monomer (CII_M_), high molecular weight forms of CII (CII_hmw_), and the SDHA subunit of CII; dashed red line, CIV dimer (CIV_D_) and supercomplexes of CIV (CIV_SC_); full black line, F_1_ subcomplex of CV (F_1_Sub.) and monomer of CV (CV_M_) based on the signals of SDHA (C), Cox1 (D), and CV-α (E) subunits.

To follow the potential differences between cultured cells and tissues, we examined the stability of CII_hmw_ from rat heart mitochondria in the presence of CBG. While we detected a complete breakdown of CII_hmw_ in the CNE gel after the addition of CBG to the sample (Figure 6A), most of the CII_hmw_ was unaffected under 2D CNE/CNE_CBG_ conditions. Based on good reproducibility of the experiments, we can conclude that CII_hmw_ do have higher MW and are more stable in tissues than in cultured cells. This may indicate that CII has different interaction partners in tissues and cultured cells, and CII_hmw_ thus ultimately represents several structurally and functionally different SCs. As in cultured cells, CIV and its SCs were unaffected (Figure 6A, 6D), while CV partially dissociated from its higher forms to the monomeric and the F_1_ sub-complex forms (Figure 6E). The sensitivity of CII and CV to CBG indicates a similar type of mild interactions responsible for the formation of their respective higher molecular weight complexes.

### CII co-immunoprecipitates with CV

**Figure 7:**
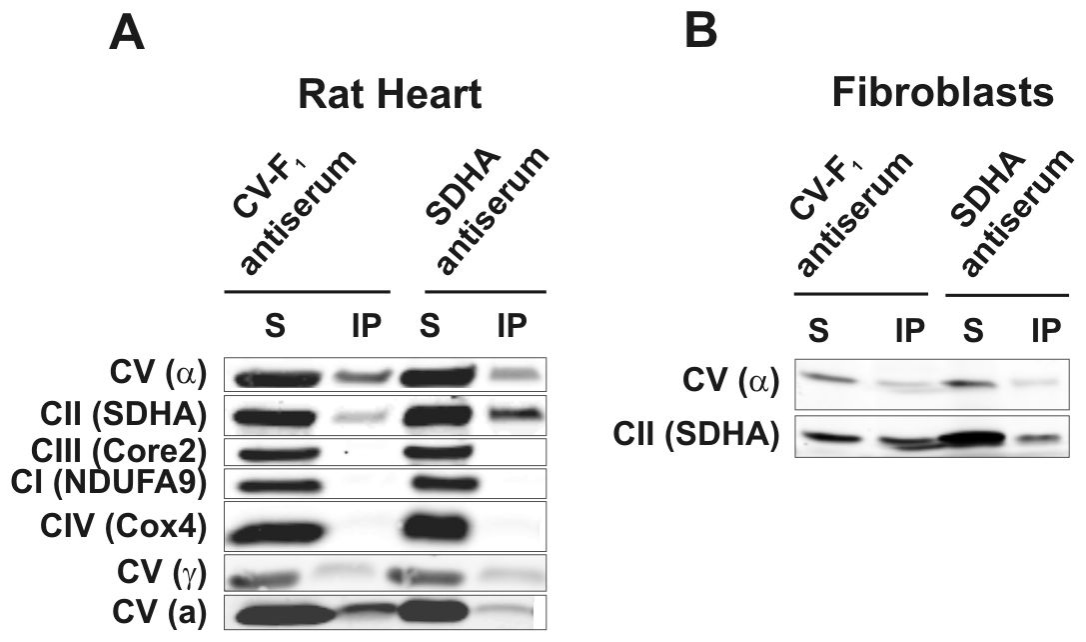
CII and CV co-immunoprecipitates. After digitonin solubilisation of mitochondria from the rat heart (A) and human fibroblasts (B), CV was immunoprecipitated with antibodies to its F_1_ part and CII with anti-SDHA. Proteins in the immunoprecipitate (IP) and solubilisate (S) were separated by SDS PAGE and individual subunits of OXPHOS complexes detected as indicated with antibodies to CI, NDUFA9; CII, SDHA; CIII, Core2; CIV, Cox4; CV, α, γ, a.

To investigate possible interactions between CII and CV by a different approach, we immunoprecipitated CV from rat heart mitochondria (Figure 7A) using a highly specific rabbit polyclonal antibody (CV–F_1_) to CV subunits α, β and γ. The antibody immobilised to agarose beads immunoprecipitated whole CV, as evidenced by the presence of both F_1_ (α and γ) and F_o_ (a) subunits. The immunoprecipitate was free of CI, CIII and CIV subunits, but it contained a significant amount of the SDHA subunit of CII. Similarly, SDHA was co-immunoprecipitated using CV-F_1_ antibody and solubilised fibroblasts (Figure 7B). In a cross-experiment, we immunoprecipitated CII from heart mitochondria using a highly specific monoclonal antibody against SDHA. The resulting immunoprecipitate contained CII as well as the whole CV as revealed by the presence of subunits from F_1_ and F_o_ parts of CV (Figure 7A). In contrast, it was free of CI, CIII and CIV. Again, CV was also co-immunoprecipitated using SDHA antibody and solubilised fibroblasts (Figure 7B). As none of the commercially available antibodies against SDHC and SDHD we tested were reasonably specific, we cannot confirm the presence of fully assembled CII in the precipitate. In principle, it is possible that only the two hydrophilic subunits SDHA and SDHB are present in the SC with CV. Notwithstanding, this result is compatible with recent data showing the presence of CII as well as CV in the mitoK_ATP_ channel complex, whose size was found to be approximately 940 kDa, similarly to CII_hmw_ forms observed in tissues.

### CII does not form SC with TCA cycle enzymes

**Figure 8:**
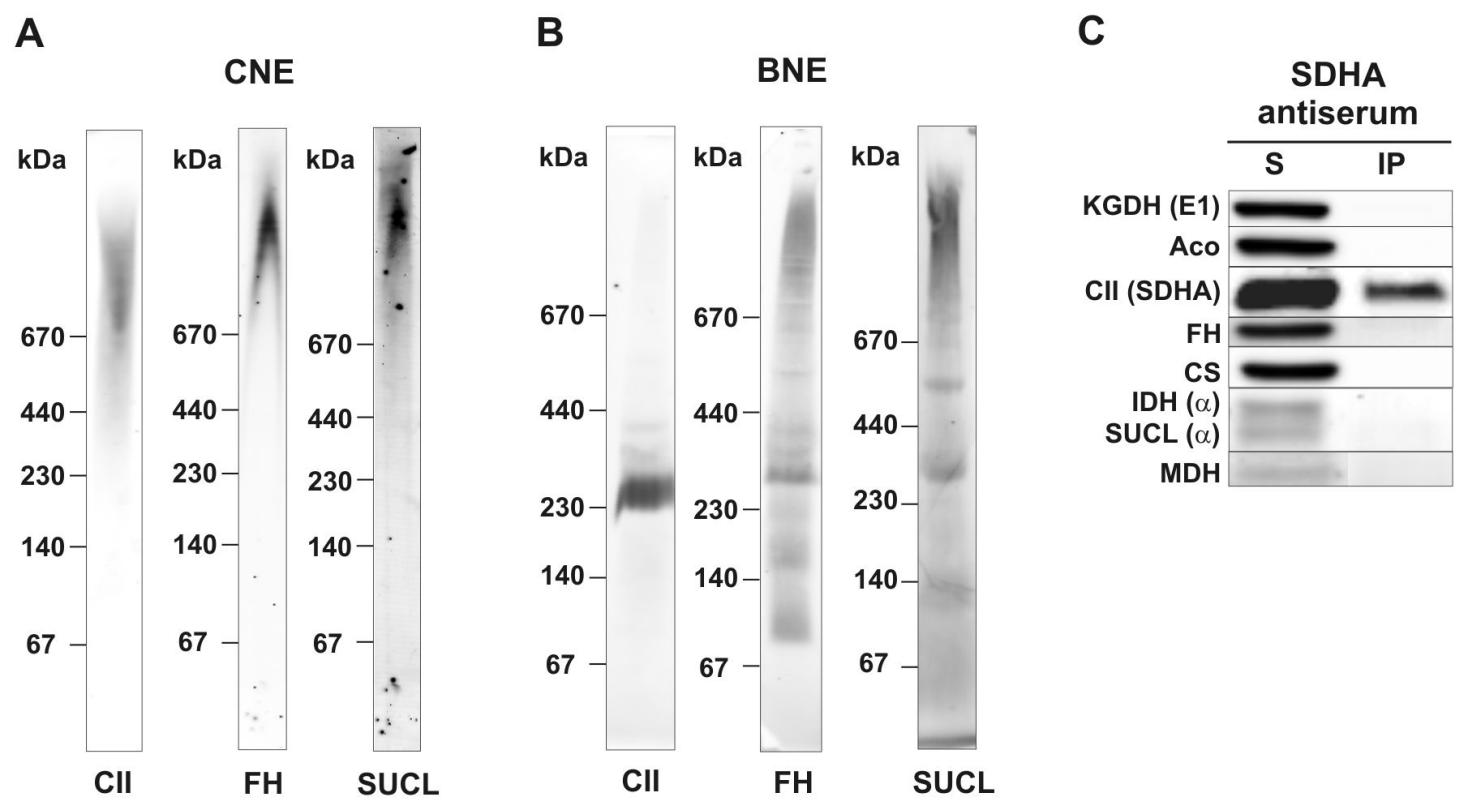
CII does not associate with other components of TCA cycle. (A) Mitochondrial membrane proteins from rat heart were solubilised with digitonin (4 g/g protein) and 20 µg protein aliquots separated using CNE. SDHA subunit of CII (CII), fumarase (FH) and subunit α of succinyl-CoA synthetase (SUCL) were detected with specific antibodies. (B) After digitonin solubilisation of mitochondria from rat heart, CII was immunoprecipitated with anti-SDHA antibody (SDHA antiserum). Proteins in the immunoprecipitate (IP) and solubilisate (S) were separated by SDS PAGE and analysed for the presence of individual components of the TCA cycle: α-ketoglutarate dehydrogenase subunit E1, KGDH (E1); aconitase, Aco; CII, SDHA; fumarase, FH; citrate synthase, CS; isocitrate dehydrogenase subunit α, IDH (α); succinyl-CoA synthetase (subunit α), SUCL (α); malate dehydrogenase, MDH.

CII can also potentially interact with components of the TCA cycle. We therefore used CNE to separate digitonin-solubilised (4 g/g) rat heart mitochondria and subsequently analysed the lysate by western blotting for the presence and distribution pattern of individual TCA cycle enzymes, which were then compared with the distribution of CII. We observed high molecular weight form complexes of fumarase and succinyl-CoA synthetase in the region above 670 kDa (Figure 8A). Although they dissociated into lower molecular forms after the addition of CBG, as was the case for CII (Figure 8B), the CNE migration pattern for both fumarase and succinyl-CoA synthetase was slightly different from that of CII, which does not support the existence of their direct interaction. Other digitonin-solubilised TCA cycle enzymes did not show any co-migration with CII on the CNE gels (not shown).

To check for the potential associations between CII and other TCA cycle enzymes, we immunoprecipitated CII from rat heart mitochondria using the monoclonal antibody against the SDHA subunit as above. The resulting immunoprecipitated CII contained no other TCA cycle enzymes, namely α-ketoglutarate dehydrogenase (subunit E1), aconitase, fumarase, citrate synthase, isocitrate dehydrogenase (subunit α), succinyl-CoA synthetase (subunit α) or malate dehydrogenase (Figure 8B).

## Discussion

The key finding of this study is the discovery of CII propensity to form higher molecular structures (CII_hmw_) in the IMM. We demonstrated that under sufficiently mild conditions, CII associates into CII_hmw_ forms in both mammalian cultured cells and tissues. As the representative cell line/tissue we used human fibroblasts and rat heart, and we have clearly shown that CII_hmw_ are present regardless of the species (rat, mouse, human), tissue type (heart, liver, brown adipose tissue) or the origin of the cell line (fibroblasts, kidney cells). As such, CII_hmw_ can be found in mitochondria with a wide range of content of the respiratory chain complexes. The interactions responsible for CII_hmw_ formation must be rather weak as the supramolecular structures are not retained under the conditions of the commonly used native electrophoretic techniques, such as BNE or hrCNE [26,28], where either negatively charged CBG or additional detergents are present and, presumably, disrupt the weak interactions responsible for CII_hmw_ formation. Thus, these complexes can only be visualised using the CNE electrophoretic system, where proteins migrate according to their intrinsic charge routinely lost by the charged dyes or detergents used to introduce the net charge to the protein micelles formed during the solubilisation of the membrane. Despite the lower resolution of CNE in comparison with other native electrophoretic systems [31], we have shown that CII_hmw_ forms differ in their apparent molecular mass between tissues (500 – over 1000 kDa) and cultured cells (400–670 kDa). The reasons for this difference are not immediately obvious. Possibly, this may be the effect of detergent (i.e. digitonin) and its concentrations used for solubilisation of proteins from the IMM. Apart from the critical micellar concentration [32], the ratio of the detergent and the protein can also dictate the outcome of the solubilisation process. As tissues display higher density of mitochondria than cultured cells, the use of the same detergent/protein ratio for both may yield different results when resolving the mitochondrial SCs. Another possible reason for the different mobility of CII_hmw_ from the two sources could be a different phospholipid composition of the IMM between cells and tissues, although the recent work indicates similarities in the relative abundance of mitochondrial phospholipids in tissues and cultured cells [33,34]. Ultimately, this difference may simply reflect a higher number of OXPHOS complexes in the IMM [33,35] and different energetic demands of tissues when compared with cells that lead to a higher probability of CII uptake into larger structures in the tissue mitochondria. Importantly, the observed differences between cells and tissues in CII_hmw_ size and stability as well as their dependence on mtDNA depletion, support the view that they reflect biological properties of complex II and do not represent an artefact of CNE electrophoresis.

When assessing the stability of CII_hmw_ complexes, we detected the CII_hmw_ as the dominant structural form of CII in digitonin solubilisates under the CNE separation conditions. The presence of either detergents (n-dodecyl-β-D-maltoside, deoxycholic acid) or CBG in the running buffer, i.e. conditions usually used to achieve better separation and resolution in the hrCNE and BNE electrophoretic systems, readily dissociated CII_hmw_ in both cells and tissues to CII monomers and individual subunits. Even the very low CBG concentration added to the sample (0.02%, 50-fold less than used in BNE samples) was sufficient for the complete CII_hmw_ dissociation. Similarly, incubation of the CNE gel slice with separated CII_hmw_ in a CBG solution also caused a partial dissociation of the CII_hmw_ structures, mainly in cultured cells. The addition of CBG to the sample/solution induces a dissociation effect by binding of the dye to proteins surface and introduction of a negative charge that can affect intermolecular interactions. Apparently, this process is more effective in solution than in the gel slice.

To understand the CII_hmw_ function, it is important to define CII interaction partners in CII_hmw_. The most obvious candidates would be other OXPHOS complexes, but the putative presence of CII in the SCs with other OXPHOS complexes is still a matter of discussion. For example, single particle electron microscopy and X-ray imaging structural studies seem to contradict such idea [36,37]. These methods did not reveal the presence of CII in any type of SCs. It should be noted, though, that due to its relatively small size, CII may simply be below the detection limit of these techniques. Similarly, studies of the assembly kinetics of the CI+CIII+CIV SC did not reveal any participation of CII in this process [38]. On the other hand, at least some immunocapture and electrophoretic experiments demonstrated the existence of a large respirasome comprising respiratory chain complexes, including CII, as well as mobile electron carriers [17]. We therefore attempted to find any indication of the interaction between CII and other OXPHOS complexes. CNE analysis and in-gel activity staining in CNE gels pointed to a possible interaction with CIV or CV, but due to the low resolution of protein bands in the CNE gels it is hard to interpret this as genuine interactions. In the MW range 400–670 kDa where CII_hmw_ are found in the cells, many other protein complexes and small SCs migrate. It is more likely that CII_hmw_ represents CII oligomers co-migrating with CV or CIV_D_, as incorporation of CII into other complexes migrating in this range would require a shift in the electrophoretic mobility of the resulting SCs towards MW greater by at least 140 kDa, i.e. the molecular weight of the CII monomer. On the other hand, CII_hmw_ in the tissues with size over 1 MDa may represent CII as a part of OXPHOS SCs.

In another attempt to detect specific OXPHOS interacting partner(s) for CII, we studied cell lines with both isolated and combined deficiencies of OXPHOS complexes. With one of the interaction partners missing, the CII_hmw_ signal would be decreased or undetectable in the respective cell line. Such interdependency is well described for canonical OXPHOS SCs [12,17,38]. However, in our experiments, the levels and position of CII_hmw_ appear to be unchanged in cells with isolated defects of CI, CIV or CV, presenting additional evidence that no stable interaction is formed between CII and other OXPHOS complexes. On the other hand, when we analysed ρ^0^ cells lacking mtDNA, unassembled subunits predominated over the CII monomer, and the CII_hmw_ structures were almost absent. The absence of the mtDNA-encoded subunits impedes the assembly and function of OXPHOS in ρ^0^ cells [39]. Because CII is entirely encoded by the nuclear DNA, it was considered to be unchanged, but a recent study by Mueller et al. [40] reports a decreased level of CII with its activity reduced to 12%. Although the synthesis of the nuclear encoded subunits is unaffected in ρ^0^ cells, mitochondrial protein import is disturbed as a consequence of decreased levels of ATP and the Tim44 protein, an essential effector of mitochondrial protein import [41]. Our experiments suggest that the CII and CII_hmw_ assembly depends on fully active mitochondria and the OXPHOS complexes of the IMM.

Immunoprecipitation was another independent approach we used to detect possible CII interaction partners. Here we identified CV as a plausible interaction partner of CII both in cultured cells and tissues. Generally, immunoprecipitation is more sensitive and selective than electrophoresis and can reveal rather weak interactions. Based on our experiments, we can conclude that CII, at least partially, co-immunoprecipitates with CV, constituting possibly a part of the mitoK_ATP_ channel described in recent studies. Although the role for CII in mitoK_ATP_ remains elusive, it was shown that SDH inhibitors modulate the channel activity and subsequently the process of ischemic preconditioning [18,42]. Only 0.4% of the CII present in mitochondria is necessary to activate the mitoK_ATP_ and the inhibition of such a small portion of CII has no effect on the overall CII activity in OXPHOS [43]. The interaction of CII with CV is also not in conflict with our results with ρ^0^ cells, as CV is assembled in ρ^0^ cells, except for the two mtDNA-encoded subunits, ATP6 and ATP8 [44]. Such form of CV (lacking the two subunits) is sufficient for the survival of ρ^0^ cells as it can hydrolyse ATP produced by glycolysis and allow for the maintenance of the transmembrane H^+^ gradient by the electrogenic exchange of ATP for ADP by adenine nucleotide translocator [45,46].

An attractive proposal may be that CII may interact with other proteins from the TCA cycle, forming an organised multi-enzyme cluster. As the only membrane bound component of the TCA metabolism, CII would represent an anchor for the docking of TCA metabolism to the IMM into the spatial proximity of OXPHOS, in accordance with the known association of soluble TCA cycle enzymes with the mitochondrial membrane [47]. The existence of the metabolon composed of at least several TCA cycle proteins has been suggested [47,48]. However, most of the evidence points to the interactions of malate dehydrogenase, citrate synthase and, potentially, aconitase [19,20]. To date, no interaction involving CII has been demonstrated. Native electrophoretic systems represent a plausible model to study such interactions; although they involve solubilisation of membrane proteins by detergents, the solubilisates of whole mitochondria contain also matrix proteins. Naturally, any such interactions may be disrupted during the analysis. Here, crosslinking may help to capture such interactions in the future studies. Despite the fact that we did not identify any interacting partners for CII among the tested TCA cycle proteins, this deserves additional work as such interactions would appear functionally plausible.

It is possible that interactions of CII other than the one detected with CV do exist, and that such interactions may not even necessarily involve the whole of CII. For example, Gebert et al. [49] have published that the Sdh3 subunit of yeast CII (SDHC in mammals) has a dual function in mitochondria. It acts as a structural and functional subunit of CII and also plays a role in the biogenesis and assembly of the TIM22 complex via a direct interaction between Sdh3 and Tim18. Therefore, we cannot exclude that other CII subunits would have specific functions outside of the OXPHOS system. Notwithstanding these potential interactions of CII, our data lead to the conclusion that CII does form high molecular weight assemblies, but these structures are unlikely to represent traditional respiratory supercomplexes with CI, CIII and CIV as proposed previously by Acin-Perez et al. [17]. At least, some of these interactions are complexes with CV where CII plays a role as a regulatory component of mitoK_ATP_ channel [42]. To summarise, our findings are consistent with the emerging notion that the individual OXPHOS complexes, or they subunits, have a role that may go beyond direct involvement in the mitochondrial bioenergetics.
